# Successful Treatment of Focal Segmental Glomerulosclerosis after Kidney Transplantation with Plasma Exchange and Abatacept in a Patient with Juvenile Rheumatoid Arthritis

**DOI:** 10.1155/2016/7137584

**Published:** 2016-03-20

**Authors:** Hannelore Sprenger-Mähr, Emanuel Zitt, Afschin Soleiman, Karl Lhotta

**Affiliations:** ^1^Department of Nephrology and Dialysis, Academic Teaching Hospital Feldkirch, 6800 Feldkirch, Austria; ^2^Vorarlberg Institute for Vascular Investigation and Treatment, Academic Teaching Hospital Feldkirch, 6800 Feldkirch, Austria; ^3^Clinical Pathology and Cytodiagnostics, 6060 Hall in Tirol, Austria

## Abstract

Recurrent focal segmental glomerulosclerosis (FSGS) after renal transplantation is difficult to treat. Recently a series of four patients unresponsive to plasma exchange (PE) and rituximab, who were successfully treated with abatacept, has been reported. We present a 26-year-old Caucasian patient who suffered from juvenile rheumatoid arthritis and developed severe proteinuria eleven days after transplantation. An allograft biopsy was suggestive of recurrent focal segmental glomerulosclerosis. He did not respond to PE therapy. A first dose of abatacept produced partial remission. Four weeks later proteinuria again increased and a second biopsy showed progression of disease. After another ineffective course of PE he was given a second dose of abatacept, which was followed by rapid, complete, and sustained resolution of proteinuria. This treatment caused a significant increase in BK and JC viremia. Whether abatacept ameliorated proteinuria via an effect on podocytes or on the patient's primary disease remains speculative.

## 1. Introduction

Primary focal segmental glomerulosclerosis (FSGS) is a common cause of nephrotic syndrome and leads to end-stage renal disease in approximately 40% of cases. Recurrence after kidney transplantation occurs in 20% to 50% of patients and has been associated with decreased allograft survival [1]. The pathogenesis of primary FSGS is incompletely understood. Recent evidence suggests that immune cell dysfunction and subsequent secretion of a circulating permeability factor and podocyte maladaptation play major roles [2]. Despite ample evidence of the existence of a permeability factor, its clear identification is still lacking. Wei et al. recently provided evidence that soluble urokinase plasminogen activator receptor (suPAR) could be the circulating factor that causes FSGS [3]. Other candidates for the permeability factor are cardiotrophin-like cytokine factor-1 (CLCF-1) [4] and autoantibodies against the costimulatory molecule CD40 [5].

SuPAR probably causes podocyte damage and proteinuria by inducing podocyte B7-1 (CD80) expression, which leads to podocyte migration through inactivation of *β*1-integrin [3, 6]. This pathophysiological concept provided the framework for a B7-1-targeted therapy in FSGS.

## 2. Case Presentation

A 26-year-old Caucasian man with end-stage renal disease received kidney transplantation from a deceased donor in October 2013 after having performed chronic peritoneal dialysis and hemodialysis for seven years. He had suffered from juvenile rheumatoid arthritis since early childhood. Because the patient had initially been admitted with end-stage renal disease, his primary renal diagnosis was unknown, but secondary amyloidosis due to rheumatic disease was suspected.

**Figure 1:**
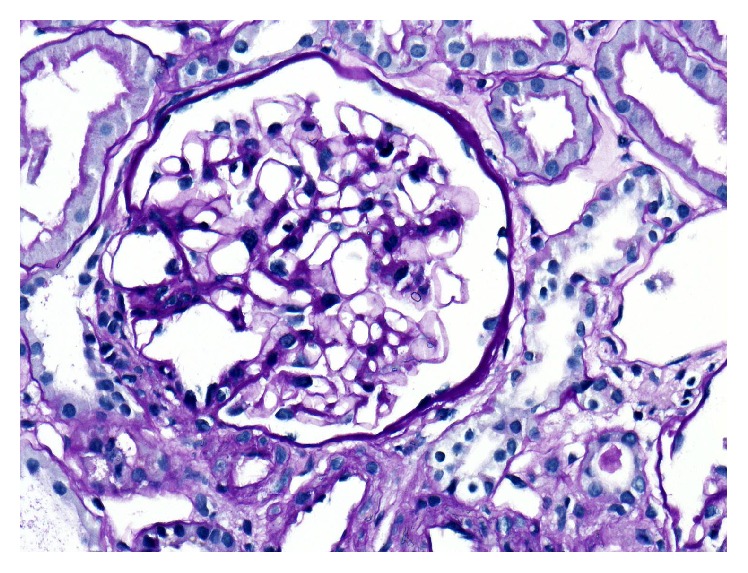
Light microscopy of the second biopsy shows mild mesangial matrix expansion and increase in mesangial cell number with focal accentuation (PAS, 200x).

The patient received induction therapy with basiliximab followed by an immunosuppressive regimen consisting of tacrolimus, mycophenolate mofetil (MMF), and prednisolone. Because of CMV mismatch he also received valganciclovir prophylaxis. He was discharged ten days after transplantation with serum creatinine of 83 *μ*mol/L. On day 11 after transplantation pronounced proteinuria with a protein-creatinine ratio of 3.34 g/g was noted. An allograft biopsy showed ten normal glomeruli with negative immunohistology, and early recurrent primary FSGS was deemed to be the most likely diagnosis. Despite nine PE sessions with exchange of one plasma volume each (5% human albumin as substitution fluid) over a period of three weeks, proteinuria remained significantly elevated after an initial decline from 5.5 g/g to around 3.5 g/g. Treatment with rituximab was not considered a therapeutic option because of severe immunoglobulin deficiency (IgG 370 mg/dL). Instead, after extensive discussion with the patient, a single dose of abatacept 10 mg per kg body weight was given. In order to avoid overimmunosuppression the MMF dose was reduced from 1000 mg to 500 mg daily. Within the next three weeks, proteinuria decreased to 1.5 g/g creatinine but over another three weeks began to rise again until it peaked at 4.5 g/g. The patient's serum creatinine increased to 151 *μ*mol/L. A second allograft biopsy showed progressive disease with diffuse mesangial expansion (Figure 1).

**Figure 2:**
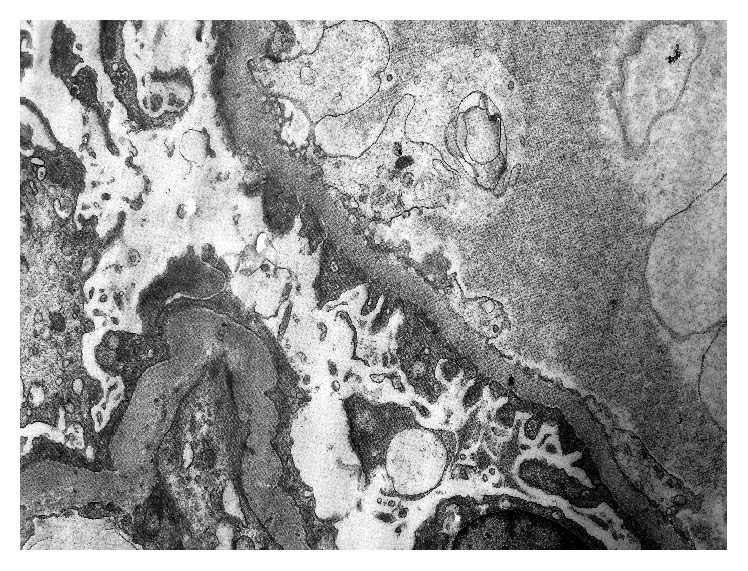
Electron microscopy of the renal biopsy reveals partial effacement and flattening of podocyte foot processes. The glomerular basement membrane is normal. No immune complex deposits are detected (4000x).

Electron microscopy revealed dystrophic podocytes with flattened foot processes (Figure 2).

**Figure 3:**
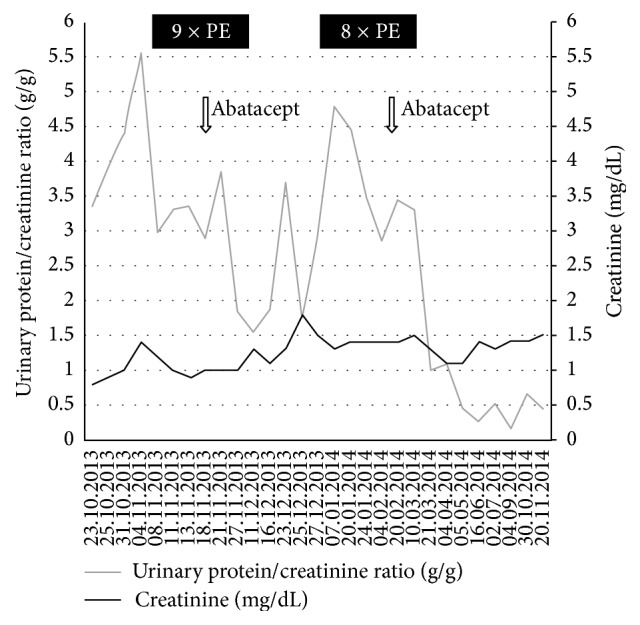
Time course of serum creatinine and proteinuria in relation to therapeutic interventions.

The patient was treated with further eight PEs, which reduced proteinuria to around 3.0 g/g creatinine with a tendency to increase. We therefore decided to give a second dose of abatacept. This was followed by a rapid decline in proteinuria to 1.0 g/g and a further decline to 0.15 g/g in the following months. The time course of treatment with PE and abatacept, serum creatinine, and proteinuria is shown in Figure 3.

Two weeks after the second dose of abatacept BK viremia was detected. BK viremia increased to 200.000 copies/mL and JC viremia (6800 copies/mL) was also detected. We discontinued MMF and reduced the tacrolimus dose, aiming for trough levels between 3 and 5 ng/mL, and prednisolone to 5 mg daily. JC viremia subsided after five months, whereas mild BK viremia (10.000 copies/mL) persisted. As the patient had negative EBV serology, regular EBV DNA monitoring was performed, which remained negative throughout the disease course. Over the next year, the patient remained in complete remission without proteinuria and with serum creatinine stable at 125 *μ*mol/L.

## 3. Discussion

Recurrence of primary FSGS after transplantation poses a serious threat to allograft function. Current therapeutic strategies such as high-dose cyclosporine A, cyclophosphamide, PE, and rituximab will induce a remission in only up to 60% of patients [2, 7, 8]. Those patients who do not respond to treatment usually progress to allograft failure. Therefore, a recent report on four patients with refractory recurrent FSGS, who showed a rapid response to abatacept, was highly welcome [9, 10]. Abatacept is a fusion molecule of a modified cytotoxic T lymphocyte-associated antigen 4 (CTLA-4) extracellular domain and a constant-region fragment of human IgG1 [11]. Abatacept and its sister molecule belatacept bind to B7-1 and B7-2 on antigen-presenting cells, thereby blocking T cell activation, and are currently licensed for the treatment of rheumatoid arthritis and the prevention of rejection in kidney transplantation [12–14]. The rationale for the use of abatacept in recurrent FSGS was the observation that B7-1 is expressed de novo on podocytes in proteinuric kidney diseases [6]. B7-1 causes inactivation of *β*1-integrin via competition between its cytoplasmic tail and talin for binding to *β*1-integrin [9]. Inactivation of *β*1-integrin will subsequently cause detachment of foot processes and proteinuria.

The enthusiasm elicited by the first report was rapidly dampened by a letter describing treatment failure of belatacept in five patients with recurrent FSGS [15]. In addition, three further patients, who did not respond to abatacept, were recently reported [16]. This case series also included a patient with minimal change disease, who experienced a marked but only transient reduction of proteinuria after abatacept. Very recently another case series of nine patients with recurrent FSGS after transplantation reported no effect of abatacept or belatacept on proteinuria [17]. It has also been questioned whether podocytes of patients suffering from FSGS or minimal change disease do actually express B7-1, which questions any rationale for B7-1 blockade in proteinuric diseases [17, 18].

Our case includes several interesting aspects. First, the primary renal disease was unknown and the diagnosis of FSGS recurrence came as a surprise. It is possible that there is a link between our patient's renal disease and juvenile rheumatoid arthritis, although such an association has not been described. Abatacept is effective in and approved for the treatment of juvenile rheumatoid arthritis [19]. We therefore cannot rule out that abatacept worked in our patient not by targeting B7-1 on podocytes but by affecting his rheumatic disease. One could imagine that cytokines or other soluble factors produced in rheumatoid arthritis (possibly CLCF-1?) may have been an important cofactor in the pathogenesis of our patient's renal disease and that reduction of these factors by targeting the rheumatic disease may have contributed to remission of proteinuria. Abatacept may also interfere with the effects of CLCF-1 or anti-CD40 autoantibodies and thereby induce remission in FSGS. Another explanation for disease remission would be that abatacept was ineffective and that our patient actually responded, albeit, somehow, delayed, to PE.

It is difficult to know why some patients seem to respond to abatacept while the majority of patients do not. The pathophysiological mechanisms underlying FSGS may be different from case to case, ranging from nonspecific podocyte injury to abnormal immune response (probably in our patient) with production of CLCF-1 or anti-CD40 antibodies and suPAR and up to podocyte expression of molecules including B7-1, B7-2, and CD40.

Following unsuccessful PE therapy, our next choice would have been rituximab.

Our patient's very low serum immunoglobulin levels made us reluctant to prescribe a therapy that might further impair the humoral immune response. We also excluded other treatment options such as high-dose cyclosporine A and steroids or cyclophosphamide because of their potential to cause severe side effects.

Although abatacept's package insert clearly excludes an increased risk for lymphoma in rheumatoid arthritis patients, the situation may be different when it is used in combination with calcineurin inhibitors and MMF. Treatment with belatacept is associated with an increased risk for lymphoma in EBV-negative renal allograft recipients [13]. Our patient had negative EBV serology and therefore belatacept would have been contraindicated. The donor's EBV status was unknown. We discussed our concerns extensively with the patient and decided to monitor EBV DNA regularly. Fortunately, we were unable to detect EBV DNA at any time.

In contrast to previous reports, we found BK virus and JC virus reactivation in our patient following abatacept therapy. To avoid overimmunosuppression we had already halved the MMF dose at initiation of abatacept. Only few data are available on virus replication and virus infection in patients receiving CTLA-4-immunoglobulins. A single-center study compared the incidence of BK virus and JC virus infection in 62 de novo kidney transplant patients, who were enrolled in the BENEFIT studies and received either belatacept or cyclosporine [20]. BK viremia occurred in 4.7% of patients in the belatacept group and in 5% of those in the cyclosporine A group. All five cases of JC virus reactivation were observed in the belatacept group. In our patient JC viremia disappeared and BK viremia decreased significantly after reducing immunosuppression, and the viral infections had no negative effects on the patient's allograft and nervous system. Nevertheless, we feel that the risk of viral infection in kidney transplant recipients treated with abatacept on top of full immunosuppression and plasma exchange is a matter of concern. Careful monitoring and judicious management of immunosuppressive therapy are warranted.
